# Construction of a Competitive Endogenous RNA Network for Pancreatic Adenocarcinoma Based on Weighted Gene Co-expression Network Analysis and a Prognosis Model

**DOI:** 10.3389/fbioe.2020.00515

**Published:** 2020-05-28

**Authors:** Jing Wang, Jinzhu Xiang, Xueling Li

**Affiliations:** State Key Laboratory of Reproductive Regulation and Breeding of Grassland Livestock, School of Life Sciences, Inner Mongolia University, Hohhot, China

**Keywords:** the cancer genome atlas, pancreatic adenocarcinoma, weighted gene co-expression network analysis, prognostic signature, competing endogenous RNA network

## Abstract

Pancreatic adenocarcinoma (PAAD) is a pancreatic disease with considerable mortality worldwide. Because of a lack of obvious symptoms at the early stage, most PAAD patients are diagnosed at the terminal stage and prognosis is usually poor. In this study, we firstly obtained RNA sequencing data of 181 patients with PAAD from The Cancer Genome Atlas (TCGA) database to identify early diagnostic biomarkers for PAAD. Survival-related mRNAs were identified using a weighted gene co-expression network analysis (WGCNA), and then a linear prognostic model of seven long non-coding RNAs (lncRNAs) was established using univariate and multivariate Cox proportional hazards regression analyses, which is verified using a time-dependent receiver operating characteristic (ROC) curve analysis. Finally, according to the survival analysis, we constructed a survival-related competing endogenous RNA (ceRNA) network. Our results showed that: (1) The upregulated genes related to cell cycle-related pathway (including homologous recombination, DNA replication and mismatch repair) in PAAD can increase the proliferation ability of cancer cells; (2) The 7-lncRNA signature can predict the overall survival (OS) of PAAD patients; and (3) The key mRNAs and lncRNAs are involved in mutual regulation in the ceRNA network.

## Introduction

Pancreatic adenocarcinoma (PAAD) is a serious pancreatic disease, pancreatic ductal adenocarcinoma (PDAC) accounts for >90% of all pancreatic cancer (Kleeff et al., 2016). Although the incidence of PAAD is very low, it is still the fourth leading cause of cancer-related death in the United States, and it is expected to become the second leading cause by 2030. The overall 5-year survival rate is about 5–7%, and the average survival time is about 6 months (Rahib et al., 2014; Siegel et al., 2018; Orth et al., 2019). There is no reliable method for screening for and early detection of PAAD, so most patients are diagnosed at an advanced stage of the disease (Tesfaye et al., 2018). PAAD has very few common genetic mutations and no clearly clinically relevant biomarkers. In this respect, the study on PAAD lags far behind other solid tumors (Gallego et al., 2017; Gallmeier and Gress, 2018). The most commonly mutated genes are KRAS (GTPase) and cyclin-dependent kinase inhibitor 2A (CDKN2A) (Caldas and Kern, 1995; Dunne and Hezel, 2015) that related to PAAD. Because of the genetic heterogeneity of pancreatic cancer, the intricate stromal microenvironment, and the complex interplay with the immune system, the disease is difficult to investigate (Krempley and Yu, 2017). To reduce mortality and improve the detection and risk classification of PAAD, early diagnostic biomarkers (Le et al., 2016) and therapeutic targets urgently need to be determined.

Long non-coding RNAs (lncRNAs) are generally defined as RNA transcripts >200 nucleotides that do not encode a polypeptide (Jathar et al., 2017), which are located in the nucleus and cytoplasm of eukaryotic cells. A large number of experimental studies have shown that some lncRNAs play an important role in the occurrence and development of malignant tumors (Yan et al., 2015; Fu et al., 2016; Li et al., 2016). MicroRNAs (miRNAs) are a kind of small RNA that play a role in gene silencing and translation inhibition by binding to target mRNAs (Ambros, 2004). The regulatory network between miRNAs and target genes is very complex, and the network can regulate most physiological activities, including tumorigenesis, metastasis and metabolism (Di Leva et al., 2014; Rupaimoole and Slack, 2017; Alamoudi et al., 2018). In recent years, a competing endogenous RNA (ceRNA) hypothesis has been proposed and developed to explain the relationships between coding genes (encoding mRNAs) and non-coding genes (encoding miRNAs, lncRNAs, circRNAs, etc.) in cells and regulation of mRNA expression. Some miRNAs can recognize and bind to microRNA response elements (MREs) in mRNAs, lncRNAs and circRNAs. Through MREs, lncRNAs act as miRNA “sponges,” leading to changes in the levels of mRNAs regulated by miRNAs (Salmena et al., 2011; Tay et al., 2014). Several studies have confirmed that the lncRNA-miRNA-mRNA ceRNA network is related to the development of many cancers (Karreth and Pandolfi, 2013; Zhou et al., 2014; Qi et al., 2015; Fang et al., 2018; Gong et al., 2019). There are similar studies in pancreatic cancer. AFAP1-AS1 promotes the growth and invasion of pancreatic cancer by upregulating the IGF1R oncogene through sequestration of miR-133a (Chen et al., 2018a). And lnc-Sox2ot promotes EMT and stemness by acting as a ceRNA in PDAC (Li et al., 2018b). These lncRNAs can also be regarded as biomarker candidates. These studies show that these lncRNAs can regulate the development of pancreatic cancer by acting as a ceRNA. Through ceRNA, we can find more biomarker candidates. However, the study on PAAD are very limited, and there is still a lack of comprehensive analysis of lncRNAs and miRNAs related to PAAD based on high-throughput sequencing and large-scale samples.

In this study, the RNA sequencing data of 181 patients with PAAD from the TCGA database were used to identify survival-related mRNAs by weighted gene co-expression network analysis (WGCNA) (Langfelder and Horvath, 2008), and a 7-lncRNAs linear prognostic signature was then established using univariate and multivariate Cox proportional hazards regression analyses and verified using a time-dependent receiver operating characteristic (ROC) curve analysis. At last, according to the survival analysis, we constructed a survival-related ceRNA network.

## Materials and Methods

### Data Acquisition and Preprocessing

RNA expression data and clinical information of PAAD patients were obtained from the TCGA database (https://portal.gdc.cancer.gov/). The RNA data of the 181 samples involved both Illumina HiSeq RNA-seq and miRNA-seq data. Among them, 150 cases of ductal and lobular neoplasms and 27 cases of adenoma and adenocarcinomas were included, and 4 cases of paracancerous samples were used as controls, and we deleted one sample without miRNA data. The clinical data used in the analyses included gender, survival status, cancer status, age (>60/ ≤ 60), grade, stage and tumor, node, metastasis (TNM) stage. All data can be downloaded free of charge. The mRNAs and lncRNAs were identified using GENCODE (https://www.gencodegenes.org/), and the mRNAs and lncRNAs not included in the database were removed. Eventually, 19600 mRNAs and 15129 lncRNAs were obtained for the subsequent analyses. The raw counts for differential analysis and the GdcVoomNormalization function of GDCRNATools (Li et al., 2018a) was used to normalize the data for WGCNA and prognostic model analysis. The raw count data were normalized using the trimmed mean of M-values (TMM) method implemented in “edgeR” (Robinson et al., 2010) and then transformed using voom in limma for subsequent analysis (Law et al., 2014). GSE62452 is obtained from GEO database (Yang et al., 2016). We downloaded the expression matrix that the author has processed, and this data set contains 65 cases with complete clinical information. We download PACA-AU data set from ICGC database (https://icgc.org/), which include 91 samples with complete clinical information.

### Analysis of Differentially Expressed mRNAs, miRNAs, and lncRNAs (DEmRNAs, DEmiRNAs and DElncRNAs)

R package “edgeR” was applied to analyze the difference between 177 cancer samples and 4 paracancerous samples. Because of the limitation of paracancerous samples, we used “upSample” and “SMOTE” in “caret” package to resample the samples and get the intersection of all results. The differentially expressed genes (DEGs) in the data set with |log2 (fold change)|≥1 and adjusted *P* ≤ 0.05 were selected for the subsequent analyses. 1152 DEmRNAs, 60 DEmiRNAs and 97 DElncRNAs were identified.

### Construction of Weighted Gene Co-expression Network of DEmRNAs

We conducted a weighted gene co-expression network analysis (WGCNA) to analyze the interaction between genes, which can describe the patterns of the gene expression profiles. WGCNA was used to evaluate the correlations between the 1152 DEmRNAs and patients' clinical information. The pairwise Pearson correlation was applied to evaluate the weighted co-expression relationship among all the data set subjects in the adjacency matrix. Average linkage hierarchical clustering was used to check whether there are outliers in 177 samples. We calculated the dissimilarity for samples dendrogram and removed some outliers. Parameter β could emphasize strong correlations between genes and penalize weak correlations. The mean connectivity and scale independence of network modules were analyzed using the gradient test under different power values, which ranged from 1 to 20. According to the scale-free topology criterion, select a soft threshold power (β) that can make the correlation of nodes in the co-expression networks get 0.9. The green module in the WGCNA analysis was related to the PAAD OS. Using the STRING database (https://string-db.org/) for a protein–protein interaction (PPI) analysis. After the weighted correlations were determined, the characteristics of the network were displayed using Cytoscape 3.7.1 (http://www.cytoscape.org/). Using the DiseaseMeth2.0 (http://bio-bigdata.hrbmu.edu.cn/diseasemeth/) for methylation analysis. *T*-test was used to analyze the methylation level of gene promoter regions (2kb upstream of TSS to 0.5 kb downstream) in cancer tissues and paracancerous tissues. Absolute Methylation Difference > 0.2 was considered as differential expression. The function of the green module genes was annotated using the “clusterProfiler” package of Bioconductor (Yu et al., 2012). Gene Ontology (GO), Kyoto Encyclopedia of Genes and Genomes (KEGG) and Gene Set Enrichment Analysis (GSEA) enrichment. GSEA is a statistical method to assess whether a priori defined set of ordered genes shows statistically significant, concordant differences between two different biological statuses (Damian and Gorfine, 2004). For each marker gene, we divided the TCGA data set into two groups according to the median of expression level. According to the differential analysis of high and low expression groups, we sorted the DEGs according to logFC, then constructed the ordered gene set and enriched with GSEA. The results were sequenced according to *P-*value. The result was based on the threshold of *P* < 0.05 and enrichment score >1.0.

### Construction of lncRNA Prognostic Signature for Predicting PAAD OS

The TCGA data set was divided into a training set (105) and a test set (72). In the training set, univariate Cox proportional hazards regression was carried out using the DElncRNAs, and those with *P* < 0.05 were selected for further analysis. A model was then selected step by step using the Akaike information criterion (AIC) to avoid overfitting; multivariate Cox proportional hazards regression analysis was performed to generate the lncRNA-based prognostic signature and we consider genes with *P* < 0.05 as independent prognostic indicators. After training and getting, the model is evaluated by leave-one-out-bootstrap cross validation (CV) in package “riskRegression” and “pec.” Then we use least absolute shrinkage and selection operator (Lasso) regression in R packages “glmnet” to verify the results (family=“cox”, maxit = 1000, nfolds = 5). The model calculated the Risk Score (RS) as follows:

$$\begin{array}{l} RS=\;\overset{n}{\underset{i=1}{\sum }}\left(\beta \;\cdot \;{expr\text{\_}level}_{i}\right) \end{array}$$

Where expr_level is the lncRNA expression and β represents the regression coefficients of lncRNAs in the multivariate Cox proportional hazards regression analysis. Patients were divided into high- and low-risk groups according to the median prognostic score. A rec eiver operating characteristic (ROC) curve analysis was then used to evaluate the performance of the prognostic signature. To further examine the relationship between lncRNAs and OS, we divided the TCGA data into a training set (75%) and a test set (25%) We trained three machine learning models (XGBoost [XGB], linear support-vector machine [SVM] and Random Forest [RF]) with 5-folds cross validation. Then, we used a ROC curve analysis to assess the performance of these classification methods.

### Construction of ceRNA Network

GDCRNATools was used to construct the ceRNA network of the three kinds of RNA. Based on the spongescan algorithm, the correlations among the expression levels of mRNAs, miRNAs and lncRNAs were explored further using five databases: TargetScan (Fromm et al., 2015), miRcode (Jeggari et al., 2012), miRTarBase (Chou et al., 2016), starBase (Li et al., 2014) and miRWalk (Sticht et al., 2018). A mRNA was considered to be a true miRNA target if the interaction was supported in TargetScan, miRTarBase, starBase and miRWalk, and an lncRNA was considered to be a true miRNA target if the interaction was supported in miRcode.

### Survival Analysis of PAAD

The prognosis model was constructed by Cox regression in package “survival,” and the result was verified by Lasso-Cox regression in package “glmnet.” To further evaluate whether a gene is related to the PAAD OS, the patient samples were divided into high-expression and low-expression groups based on the median gene expression. We used the R package “survival” and “pec” for survival analysis. Then we use “survminer” to generate Kaplan–Meier curves. Several methods (log-rank, Wilcoxon, Fleming-Harrington, Tharone-Ware and Peto-Peto) was used to compare survival time between the two groups, and *P* < 0.05 was considered to be statistically significant.

## Results

### DEmRNAs, DEmiRNAs and DElncRNAs in PAAD

**Figure 1:**
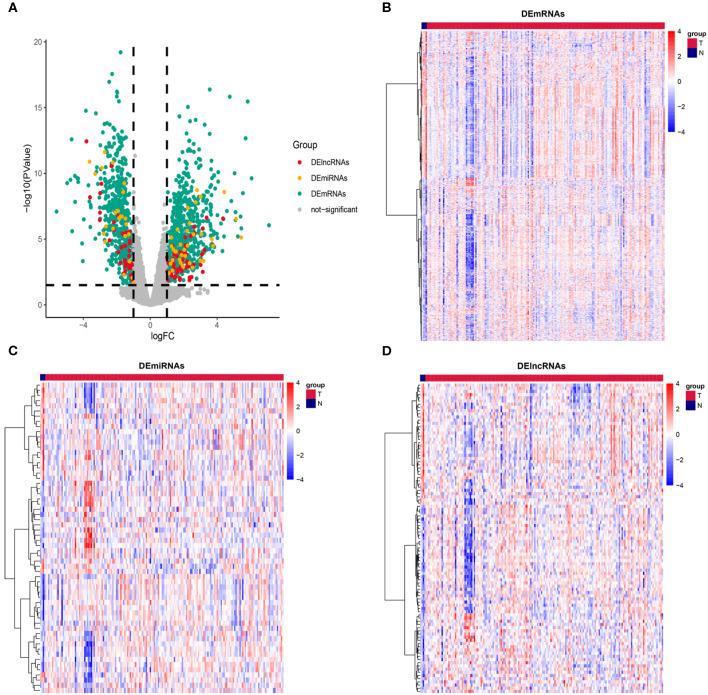
Description of DEmRNAs, DEmiRNAs, and DElncRNAs. **(A)** Volcano plot showing DEmRNAs (green), DEmiRNAs (yellow), and DElncRNAs (red), The X axis represents logFC, the Y axis represents log10 (*P* value). Genes with |logFC| ≥ 1 and *P* ≤ 0.05 were defined as DEGs. Heatmap showing the normalized expression of **(B)** DEmRNAs, **(C)** DEmiRNAs and **(D)** DElncRNAs. T indicates cancer tissue, N indicates paracancerous tissue.

The RNA data of 181 PAAD patients, involving 177 cancer samples and 4 paracancerous samples, were obtained from the TCGA database. In order to prevent the error caused by limitation of paracancerous samples, we resample the data for differential analysis between cancer tissues and paracancerous tissues. Genes with |log (fold change)| ≥ 1 and *P* ≤ 0.05 were defined as DEGs. According to this standard, we obtained 1152 DEmRNAs, comprising 611 (53.03%) upregulated genes and 541 (46.97%) downregulated genes; 60 DEmiRNAs, comprising 36 (60%) upregulated genes and 24 (40%) downregulated genes; and 97 DElncRNAs, comprising 59 (60.82%) upregulated genes and 38 (39.18%) downregulated genes (Figure 1). The complete differentially expressed RNA data are shown in Supplementary Table S1.

### Key Modules Related to PAAD OS

**Figure 2:**
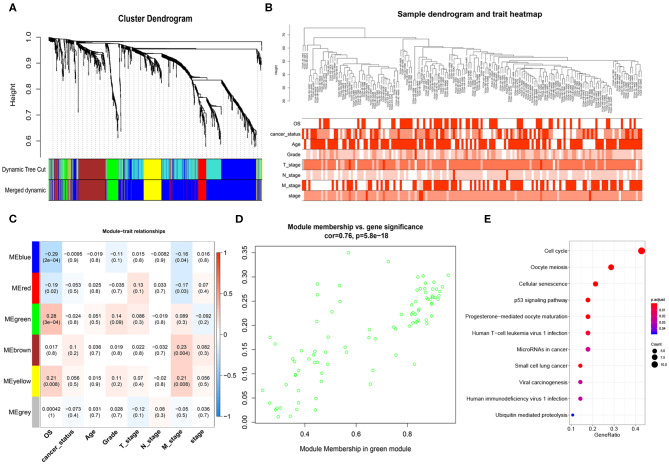
Weighted gene co-expression network analysis (WGCNA) network. **(A)** Cluster dendrogram of DEmRNAs. Each branch represents a single gene. Height indicates the Euclidean distance. Each color indicates a single module. **(B)** Hierarchical clustering tree of the TCGA-PAAD samples. Dendrogram tips are labeled with the TCGA-PAAD unique name. Height indicates the Euclidean distance. **(C)** Heatmap showing the Pearson correlation between modules and patients' clinical information. The numbers represent correlation coefficients and *P*-value. **(D)** Correlation analysis showed that genes in green module were significantly correlated with OS (cor = 0.76) **(E)** KEGG enrichment analysis of genes in the green module, ranked by *P*-value. The size of each dot represents the number of genes enriched in the pathway.

After obtaining the 1152 DEmRNAs, we used WGCNA to analyze the correlation between gene and clinical information. Hierarchical clustering was performed before analysis. We found that some samples have abnormal clustering results. We calculated the dissimilarity of samples, and found that the samples were divided into two groups (160 vs. 18 samples) near the cut-off value of 80. Some of them were far away from other samples. We considered these 18 samples as outliers and deleted them, and the remaining 160 PAAD samples were used for the subsequent analyses. The soft threshold power (β) of 5 was selected according to the scale-free topology criterion. After choosing the appropriate β (β = 5) to classify genes with similar expression profiles into gene modules, the average linkage hierarchical clustering was conducted according to the topological overlap matrix (TOM) based on dissimilarity measurement, and divide each module into different colors (Figure 2A). The heatmap showed the correlations of the modules and the patients' gender, survival status, cancer status, age (>60/ ≤ 60), grade, stage and TNM stage (Figure 2B). The highest correlation was between the green module and OS (*r* = 0.28, *P* = 3e-04) (Figure 2C). We selected the 85 genes in the green module (Figure 2D) for Kyoto Encyclopedia of Genes and Genomes (KEGG) enrichment analyses, and we found that the cell cycle, the p53 pathway and other important pathways in cancer were enriched (Figure 2E; Massague, 2004; Stracquadanio et al., 2016; McFarlane and Wakeman, 2017; Connor et al., 2019).

**Figure 3:**
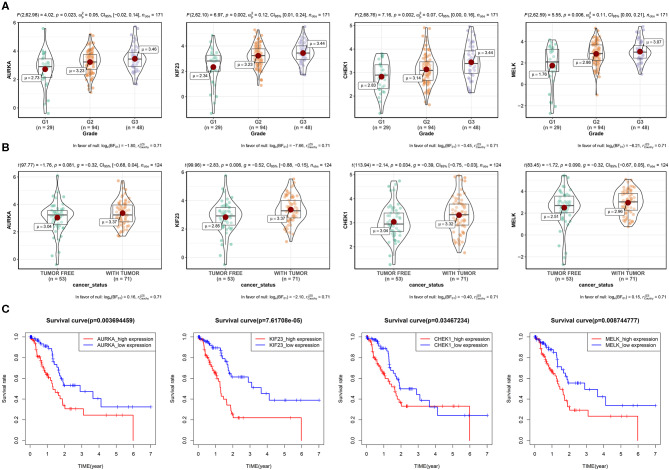
Four key genes in the green module. **(A)** Expression of four genes by grade in PAAD patients (due to the lack of expression data for G4, only the expression for G1–G3 is shown). *P*-value, 95% CI, and other information are shown at the top of the figure. **(B)** Relationships between the four genes and the cancer status among PAAD patients. **(C)** Survival analysis of the four genes. Patients were divided into high-expression and low-expression groups according to the median gene expression.

Next, we explored the protein–protein interaction (PPI) network in the green module (Supplementary Figure S1), the average connectivity (number of nodes interacting with other node in the network) in the network was found to be 29.33. Such a high degree of average connectivity suggests that these genes are likely to have synergistic effects. We performed survival analysis on the genes in the green module, and we found that four genes: maternal embryonic leucine zipper kinase (MELK), Aurora kinase A (AURKA), kinesin family 23 (KIF23) and checkpoint kinase 1 (CHEK1) were significantly related to OS (*P* < 0.05). These genes showed different expression levels in different pathological stages of PAAD (Figures 3A,B). They were at the center of the PPI network and they were also in the ceRNA network. We then explored the methylation level of the promoter regions of these genes in cancer tissues and paracancerous tissues (Supplementary Figure S2). Significant difference of methylation level in cancer tissues was found by *T*-test (*P* < 0.05). The higher the expression of these genes, the lower the methylation level, and the higher the risk for patients in the survival analysis. GSEA enrichment results showed that they are mainly related to DNA replication, mismatch repair, homologous recombination and other pathways (Supplementary Figure S3), which shows that these genes play an important role in cell division. Tumors have abnormal proliferation and abnormal cyclin kinase expression leads to uncontrolled proliferation of tumor cells, which often have germline mutations in homologous recombination repair genes, so they depend on checkpoint proteins related to DNA damage, such as CHEK1, to induce G2 block and integrate threonine kinase (ATM and ATR) signals to repair damaged DNA. The co-expression and interactions of these genes were associated with poor clinical outcomes (Figure 3C). In order to verify the prognostic value of these four key genes, we used the expression profiles of 65 cases in GSE62452 and 91 cases in ICGC with complete clinical information. The results showed that these four genes have important prognostic value (Supplementary Figure S4).

### LncRNA Prognostic Signature

**Figure 4:**
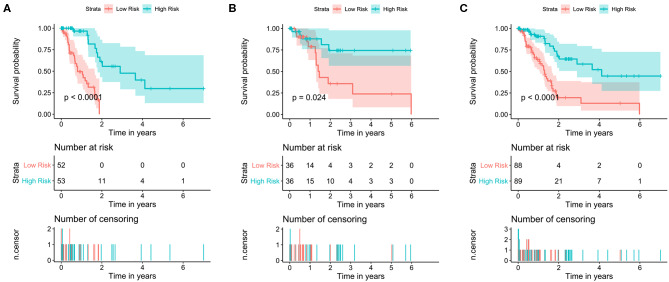
Kaplan–Meier survival curves of the training set, test set and total set. Survival curves of **(A)** training set **(B)**, test set and **(C)** total set. Patients were divided into high- and low-risk groups according to the median prognostic score. log-rank, Wilcoxon, Fleming-Harrington, Tharone-Ware, and Peto-Peto was used to compare the differences of survival curves between two groups and get consistent results (*P* < 0.0001). The *P-*value of plots based on log-rank test.

**Figure 5:**
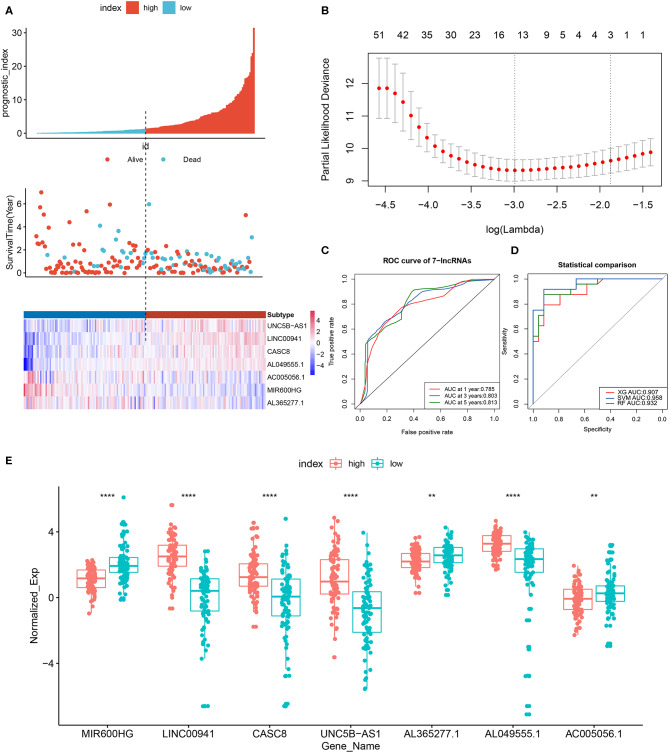
LncRNA prognostic signature model and least absolute shrinkage and selection operator (Lasso) regression results. **(A)** Patients were divided into high- and low-risk groups according to the median prognostic score. The prognostic score, OS and expression levels of 7 lncRNAs in the two groups are shown. **(B)** Regression coefficient diagram using Lasso regression. **(C)** ROC curves of the 7-lncRNA prognostic signature for predicting 1-, 3-, and 5-year survival. **(D)** Diagnostic efficiency, based on the AUC, of 7-lncRNA signature models constructed using different machine learning models (random forest, support-vector machine [SVM] and XGBoost). **(E)** Boxplot showing the expression levels of 7 lncRNAs in the high- and low-risk groups. **p < 0.01, ****p < 0.0001.

The data set was divided into training set (105) and test set (72). In the training set, the univariate Cox regression was applied and obtained 22 lncRNAs related to patients' OS (Table 1). Then these lncRNAs was used as covariates and the multivariate Cox analysis was performed to screen independent prognostic indicators, and the Akaike information criterion (AIC) was used for model selection. Finally, 7 lncRNAs was selected as PAAD independent prognostic indicators and a 7-lncRNAs prognostic model were generated. In the training process, leave-one-out-bootstrap CV was used to verify the model. After several times of verification, we acquired a relatively stable result. According to this model, a risk score was generated for each patient. The risk score was calculated as follows: Risk Score = (−0.6798 × expr_MIR600HG) + (0.5330 × expr_LINC00941) + (−0.3057 × expr_CASC8) + (0.3093 × expr_UNC5B-AS1) + (−0.4540 × expr_AL365277.1) + (0.6676 × expr_AL049555.1) + (−0.2785 × expr_AC005056.1). Four of these lncRNAs (MIR600HG, LINC00941, UNC5B-AS1, and AL04955.1) were shown to be independent prognostic indicators (Table 2), which is consistent with the Kaplan–Meier curve of the 7-lncRNA signature (Supplementary Figure S5). Three genes were associated with high risk of PAAD (LINC00941, UNC5B-AS1, and AL049555.1), while four genes were associated with protection from PAAD (MIR600HG, CASC8, AL365277.1, and AC005056.1). According to the median of risk score (1.226066), the data set was divided into high-risk group and low-risk group, and Kaplan-Meier curve is shown in Figure 4. The overall survival time of high-risk group is significantly lower than that of low-risk group [the median OS was 15.6 vs. 49.4 months, hazard ratio [HR] = 3.98, 95% confidence interval [CI]: 2.22–7.13, *P* < 0.0001] (Figure 5A).

**Table 1:** Results of univariate Cox proportional hazards regression.

**Characteristic**	**HR**	**95% CI for HR**	***P*-value**
MIR600HG	0.41	0.28–0.61	6.50E-06
LINC00941	1.7	1.3–2.1	4.50E-05
LINC01133	1.4	1.1–1.7	0.0015
CASC8	1.4	1.1–1.7	0.0032
UNC5B-AS1	1.3	1.1–1.6	0.0033
AC087741.1	0.74	0.6–0.91	0.0052
AC027097.1	0.41	0.21–0.77	0.0056
AC130456.2	1.2	1.1–1.4	0.0074
AC009065.2	1.4	1.1–1.8	0.009
AL365277.1	0.58	0.39–0.87	0.0092
LINC00857	1.5	1.1–2	0.011
SH3BP5-AS1	0.61	0.42–0.9	0.013
LINC01091	0.73	0.58–0.94	0.013
AL049555.1	1.8	1.1–2.7	0.013
AC005674.2	0.71	0.54–0.94	0.015
AP004608.1	0.85	0.74–0.97	0.015
AL139246.3	1.3	1–1.5	0.02
AC012368.1	0.7	0.51–0.96	0.028

**Table 2:** Results of multivariate Cox proportional hazards regression.

**Characteristic**	**HR**	**95% CI for HR**	***P*-value**	**lasso**
MIR600HG	0.51	0.31–0.84	0.008	−0.13
LINC00941	1.7	1.21–2.39	0.002	0.19
CASC8	0.74	0.49–1.10	0.139	0.06
UNC5B-AS1	1.36	1.09–1.70	0.006	0.02
AL365277.1	0.64	0.38–1.07	0.09	−0.12
AL04955.1	1.95	1.04–3.65	0.037	-
AC005056.1	0.76	0.51–1.11	0.157	-

The prognostic scores (risk scores) of patients from the PAAD data set were analyzed by the 7-lncRNA expression levels. A similar result was found using Lasso regression (the model, which involved five lncRNAs, and four of them included in the 7-lncRNA signature (MIR600HG, AL365277.1, UNC5B-AS1, and LINC00941) (Figure 5B). A time-dependent ROC curve analysis was used to evaluate the prognostic value of the 7-lncRNA signature. Based on the TCGA data set, the area under the curve (AUC) for the 7-lncRNA signature was 0.785, 0.803 and 0.813 for 1-, 3-, and 5-year survival, respectively (Figure 5C). The 7-lncRNA signature was used to build three machine learning models (random forest, SVM, and XGBoost) in training set to predict the PAAD OS. ROC curves was used to evaluate the prediction performance of the three machine learning models in test set. The results showed that the AUC values for SVM, XGB and RF were 0.958, 0.907, and 0.932, respectively (Figure 5D). The expression level of 7-lncRNA signature in high-risk group and low-risk group was significantly different (Figure 5E). After the prognostic performance in 177 patients with clinical information was evaluated by univariate Cox proportional hazards regression we found the cancer status (*P* < 0.001), grade (*P* = 0.013), stage (*P* = 0.01) and 7-lncRNA (*P* < 0.001) signature could be used as independent prognostic indicators of PAAD (Table 3, Supplementary Figure S6). We further used these clinical information as covariates to conduct a multivariate Cox proportional hazards regression analysis. The results showed that the 7-lncRNA signature (HR 2.68, *P* = 0.001) and cancer status (HR = 1.76, *P* = 0.012) were independent prognostic factors for PAAD OS.

**Table 3:** Predictive values of related clinical features and risk score.

	**Univariate analysis**			**Multivariate analysis**		
**Characteristics**	**Hazard.ratio**	**CI95**	***P*-value**	**Hazard.ratio**	**CI95**	***P-*value**
Gender	1.23	0.73–2.09	0.436	-	-	-
cancer_status	2.47	1.57–3.89	<0.0001	1.76	1.13–2.75	0.012
age	1.44	0.81–2.57	0.211	-	-	-
Grade	1.43	1.08–1.89	0.013	1.36	0.92–2.02	0.124
Stage	1.72	1.14–2.59	0.01	1.11	0.62–2.01	0.721
T_Stage	1.43	0.97–2.11	0.069	-	-	-
N_Stage	1.53	0.95–2.46	0.081	-	-	-
M_Stage	0.85	0.65–1.11	0.236	-	-	-
Risk score	3.98	2.22–7.13	<0.0001	2.68	1.47–4.89	0.001

### ceRNA Network Related to PAAD OS

**Figure 6:**
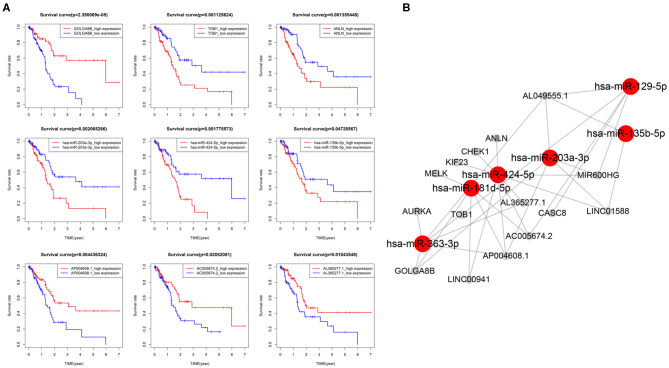
CeRNA network related to PAAD overall survival (OS). **(A)** Kaplan–Meier curves of 3 mRNAs (TOB1, GOLGA8B and ANLN),3 miRNAs (203a-3p, 424-5p, and 135b-5p) and 3 lncRNAs (AP004608.1, AC005674.2, and AL365277.1) were selected for display. **(B)** CeRNA network related to PAAD OS.

DEmRNAs, DEmiRNAs and DElncRNAs was used to construct a global ceRNA network. Unfortunately, we found that UNC5B-AS1 and AC005056.1 were not in the global ceRNA network. To simplify the network results, we used the 4 key mRNAs (AURKA, MELK, KIF23, and CHEK1) and 5 key lncRNAs (MIR600HG, LINC00941, CASC8, AL049555.1, and AC005056.1) as the center to prune the global ceRNA network, and then we obtained the key-nodes ceRNA network. We performed survival analysis on these nodes, and removed the nodes that were not significantly related to OS (Figure 6A), and we finally obtained seven mRNAs, six miRNAs, eight lncRNAs and 32 edges in the survival-related ceRNA network, including the 4 key mRNAs and 5 key lncRNAs mentioned above (Figure 6B). The survival analysis showed that upregulation of 6 mRNAs (ANLN, TOB1, AURKA, MELK, KIF23, and CHEK1), 3 miRNAs (miR-135b-5p, miR-424-5p, and miR-203a-3p) and 4 lncRNAs (AL049555.1, LINC00941, LINC01588, and CASC8) was associated with poor PAAD OS, while down regulation of the remaining 6 genes (GOLAG8B, miR-129-5p, MIR600HG, AL365277.1, AC005674.2, and AP004608.1) in PAAD indicated a longer survival time.

## Discussion

PAAD, the most common form of pancreatic cancer, is still one of the most aggressive and fatal cancers in the world (Kleeff et al., 2016). In the past, great efforts have been made to elucidate the molecular mechanism underlying the pathogenesis of PAAD at the level of coding and non-coding genes, and to find molecular targets related to PAAD. For predicting the prognosis of PAAD, molecular signatures are likely to be better than single biomarkers. Research based on a single-omics level limits deep exploration of the molecular mechanisms underlying PAAD, and each gene in the cells does not play an independent role. The concept of the ceRNA network was proposed in recent years. LncRNAs act as miRNA “sponges” to prevent miRNAs from binding to mRNAs (Salmena et al., 2011). A system analysis based on the hypothesis of the mRNA-miRNA-lncRNA ceRNA network may lead to a molecular signature with better prognostic value in PAAD than a single gene.

The multi-omics or meta-analysis has been applied to identify the biomarkers of pancreatic adenocarcinoma especially for PDAC (Chen et al., 2018a; Klett et al., 2018; Mishra et al., 2019). Mishra et al. Identified survival associated genes using multi-omics data from PDAC patients (Mishra et al., 2019). And Klett et al. identified 17 genes that were previously recognized as PDAC biomarkers (Klett et al., 2018) and several genes (B3GNT3, DMBT1, DEPDC1B) and lncRNAs (PVT1 and GATA6-AS) have not been reported in PDAC before, which is important for developing early detection and effective treatment regimens for PDAC (Mishra et al., 2019). At present, the focus of multi-omics study based on PAAD is to associate gene expression profile with other omics data (DNA methylation, Copy Number Variation [CNV]) (Raman et al., 2018; Mishra et al., 2019). However, the relationship between the mRNA-miRNA-lncRNA expression patterns is not discussed. Studies have reported the important role of ceRNA network in the tumor progression of PAAD. Chen et al. confirmed that lncRNA AFAP1-AS1 promotes the growth and invasion of PDAC by upregulating the IGF1R oncogene through sequestration of miR-133a (Chen et al., 2018a). Li et al. was also observed that tumor-derived exosomal lncRNA Sox2ot enhances EMT and stemness in PDAC by acting as ceRNA (Li et al., 2018b). These studies limited to a few specific genes, which does not take the advantage of high-throughput sequencing and large-scale samples. At the same time, each gene in ceRNA network will be subject to the interaction of other genes. It is not good to describe its regulation by focusing on a few marker genes. In this study, we report the results of multi-omics analysis based on ceRNA network. Our purpose is to explore the relationship between the mRNA-miRNA-lncRNA expression patterns. In order to identify the hub genes, we screened the hub genes from DEmRNAs and DElncRNAs by WGCNA and Cox regression. We constructed a ceRNA network based on the prognosis of patients by using key mRNAs and lncRNAs. To the best of our knowledge, this is the first study to investigate the specific ceRNA network in pancreatic cancer by two-way selection, instead of “lncRNA-miRNA-mRNA” or “mRNA-miRNA-lncRNA” order pattern. We also considered the interaction of other common target genes. Inspiringly, a novel mRNA-miRNA-lncRNA triple regulatory network was constructed and each genes in this network possessed a significant prognostic value in pancreatic cancer. And our results are still valid for other types of adenocarcinoma.

In our study, WGCNA was used to identify the green module (involving 85 genes) that was related to PAAD OS, and 4 genes (AURKA, MELK, KIF23, and CHEK1) were found specifically expressed in PAAD and played an important role in the development of PAAD, which may become the prognostic markers. AURKA belongs to the aurora kinases family, and its up-regulation in pancreatic cancer is related to the poor overall survival (Xie et al., 2017). And AURKA binds to cyclin-dependent kinases (CDKs) and a variety of other proteins, such as polo-like kinase 1 (PLK1) and Aurora kinase B (AURKB), controls the progression from S (mitosis) stage and G2 stage to M (mitosis) stage (Otto and Sicinski, 2017). KIF23 is a member of the kinesin family (KIF), which is mainly responsible for cytokinin regulation. Abnormal expression of KIF23 can cause tumorigenesis and cancer development (Li et al., 2019). AURKA and KIF23 are also downstream targets of YAP/TEAD. YAP pathway has been identified as an important prognostic marker of PDAC (Rozengurt et al., 2018). Their abnormal expression in PDAC may be related to the regulatory mechanism of YAP pathway. Zhou et al. found that MELK and KIF23 may be eigengenes related to the progression of pancreatic cancer (Zhou et al., 2018). MELK is a cell cycle-dependent protein kinase belonging to the KIN1/PAR-1/MARK family (Tassan and Le Goff, 2004), which regulates intracellular signal transduction, and thereby influences various biological processes in combination with a variety of proteins, including cell cycle, carcinogenesis, cell proliferation, and apoptosis (Jiang and Zhang, 2013). MELK is up-regulated in pancreatic cancer and other types of solid tumors and plays an important role in the formation and maintenance of tumor stem cells (Lu et al., 2019). Previous study suggested that MELK could control the migration of normal and transformed pancreatic duct cells (Chung et al., 2014). Moreover, OTSSP167, the MELK-targeting compound, inhibits the growth of various human cancers, including breast, lung, prostate and pancreatic cancers (Chung et al., 2012). Pan et al. found that CHEK1 was activated in stage IV in PDAC, which was related to tumor invasion (Pan et al., 2018). CHEK1 and cell cycle-related proteins (AURKA, etc.) have synergistic effects. It is reported that simultaneous inhibition of CHEK1 and AURKA leads to cell cycle arrest and apoptosis of cancer cells (Alcaraz-Sanabria et al., 2017). We found that abnormal expression of these 4 genes may lead to abnormal proliferation of cancer cells. We speculate these genes may have synergistic effects in cancer cells that enable them to jointly regulate the cell cycle of PAAD, which agree with the statement of the cell cycle played a vital role in PAAD (Zhou et al., 2018). We used the TCGA training data set to conduct a Cox proportional hazards regression analysis, and finally obtained a 7-lncRNA signature related to the prognosis of patients with PAAD. The 7-lncRNA signature, cancer status, grade and stage were associated with the PAAD OS by univariate Cox regression. According to multivariate Cox regression, the 7-lncRNA signature and cancer status were independent prognostic factors for OS. To further assess the prediction ability of the 7-lncRNA signature, three kinds of machine learning models (SVM, random forest, XGBoost) were constructed based, and used to predict the overall risk of patients in the TCGA data set. Although research on the roles of these lncRNAs in PAAD is very limited, some lncRNAs have been identified in the literature as biomarkers. For example, MIR600HG is a potential therapeutic target and molecular biomarker in PAAD (Song et al., 2018); LINC00941is thought to be associated with tumor cell proliferation and metastasis in gastric cancer (Liu et al., 2019), which affects genes and proteoglycans in cancer, the Hippo signaling pathway, cancer pathways, the cell cycle and leukocyte transendothelial migration (Luo et al., 2018); Hu et al. Showed that CASC8 could reduce the glycolysis of bladder cancer cells and inhibit the growth of bladder cancer cells (Hu et al., 2017). In papillary thyroid carcinoma, UNC5B-AS1 regulates the proliferation, invasion and migration of cancer cells (Guo et al., 2019). In our study, these lncRNAs were found exhibiting the abnormal expression patterns in PAAD, and the Cox regression results show that they can be used as independent prognostic indicators of PAAD. The abnormal expression of key-lncRNAs led to poor OS, which further confirmed its role as a biomarker.

In the process of constructing the ceRNA network, we performed a survival analysis of nodes in the network. The results showed that 7 mRNAs, 6 miRNAs, and 8 lncRNAs were related to the PAAD OS, among which the upregulation of 13 of them was related to poor OS and the downregulation of the other six was associated with a longer survival period among PAAD patients. We connected these potential prognostic markers through the ceRNA network. They have special expression patterns in PAAD and may play an important role in the development of PAAD. Studies have shown that the abnormal expression of miR-203a-3p, miR-129-5p, miR-424-5p, and miR-135b-5p is related to tumor development (Wu et al., 2013; Chaudhary et al., 2017; Zhang et al., 2017; Chen et al., 2018b; Qiu et al., 2019). The abnormal expression of these miRNAs in PAAD may affect the expression of the key mRNAs and effects of the key lncRNAs, which may lead to poor OS. These miRNAs may also be biomarker candidates for PAAD OS. According to the ceRNA network hypothesis, miRNAs can bind to MREs and thereby interact with mRNAs and lncRNAs and thus affect the interactions between them (Salmena et al., 2011; Tay et al., 2014), which indicates that miRNA plays a pivotal role in the ceRNA network.

In conclusion, we identified a central mRNA-miRNA-lncRNA ceRNA network consisting of 7 mRNAs, 6 miRNAs and 8 lncRNAs, and described the regulatory pattern of mRNAs and lncRNAs in PAAD through ceRNA network, we found that the abnormal expression of each hub gene in ceRNA network will lead to poor OS. At the same time, we propose a new perspective to describe the regulatory mechanism of prognostic markers in PAAD through ceRNA network, which is a good way to identify genes related to OS of PAAD. Our findings may provide clues for the development of a promising tool for predicting the OS of PAAD.

## Data Availability Statement

Publicly available datasets were analyzed in this study. The source code for this study can be found at https://github.com/nnlrl/PDAC.

## Author Contributions

JW completed the data analysis. JX and XL participated manuscript composition. All authors read and approved the final manuscript.

## Conflict of Interest

The authors declare that the research was conducted in the absence of any commercial or financial relationships that could be construed as a potential conflict of interest.
