# Supplementary material for: Construction of a Competitive Endogenous RNA Network for Pancreatic Adenocarcinoma Based on Weighted Gene Co-expression Network Analysis and a Prognosis Model
Source: Front Bioeng Biotechnol. 2020 May 28;8:515. doi: 10.3389/fbioe.2020.00515 (PMC7270201; doi:10.3389/fbioe.2020.00515)
Supplement: Supplementary file 2 [file Data_Sheet_1.docx]

Supplementary Material

# Supplementary Figures and Tables

## Supplementary Figures


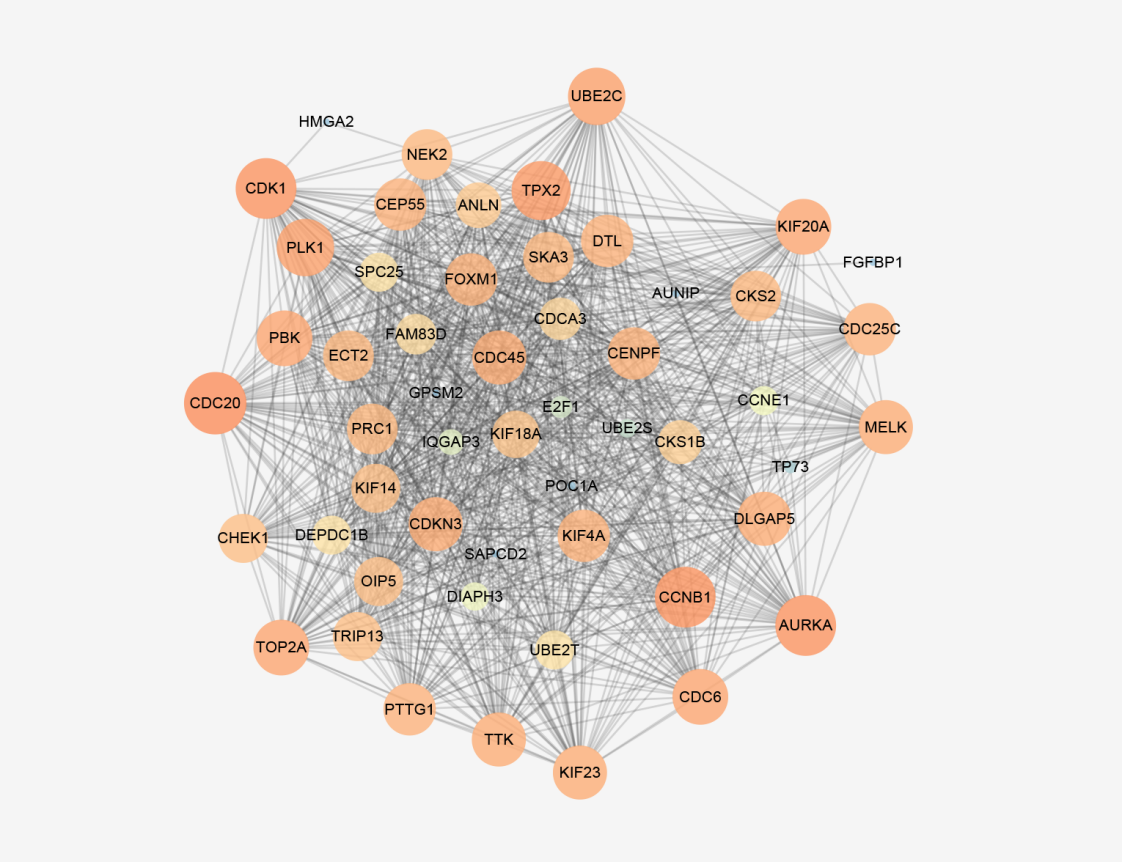


**Supplementary Figure 1 |** Protein-protein interaction network in green module

The network diagram shows the protein interaction of genes in the red module. Individual nodes (no interaction with other nodes) was removed. The color and size of nodes represent the connectivity of the genes in the network. The average connectivity of the network is 29.33 (each node has an average of 29 interactions with other nodes)


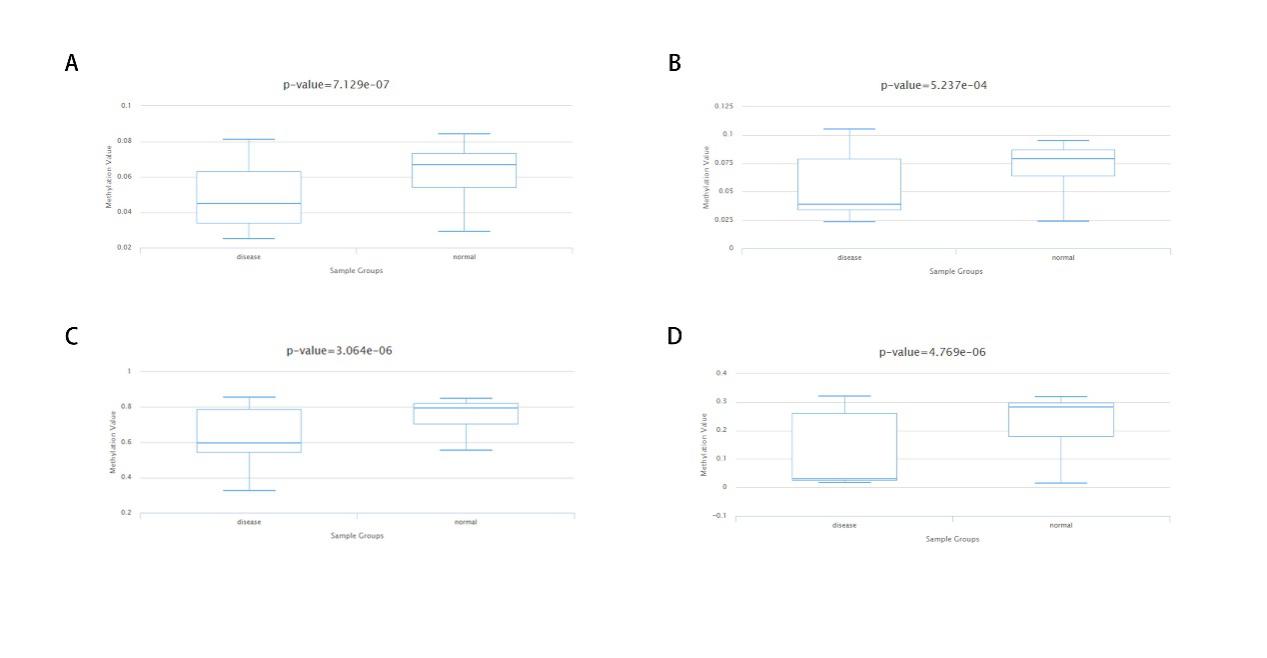


**Supplementary Figure 2 |** Description of the level of methylation of 4 key-genes

To check the methylation level of promoter region (2kb upstream from TSS to 0.5kb downstream) of 4 genes (A) AURKA, (B) KIF23, (C) CHEK1 and (D) MELK in tumor and normal tissues of PDAC, and Absolute Methylation Difference > 0.2 was considered as differential expression. P-value in the plots is obtained by t-test.


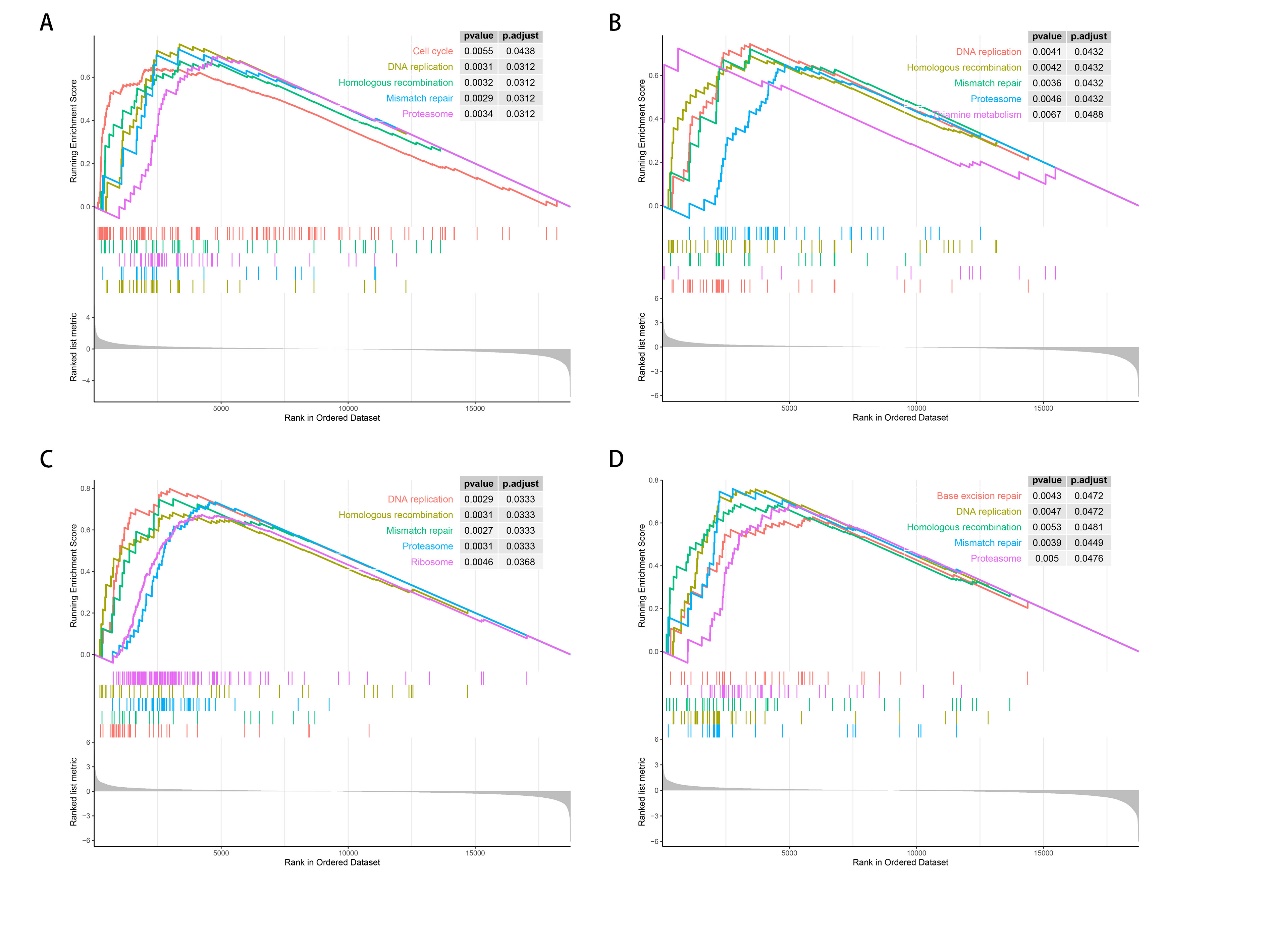


**Supplementary Figure 3 |** Description of the results of GSEA analysis of 4 key-genes

GSEA enrichment of 4 genes: (A) AURKA, (B) KIF23, (C) CHEK1 and (D) MELK. The table shows the first 5 enriched pathways (sorted by P-value). According to the median of each gene expression, the PDAC data set is divided into high expression group and low expression group. Through the differential analysis between the two groups, the gene set is sequenced and enriched by logFC. In the picture, the first 5 pathways is sequenced according to P-value


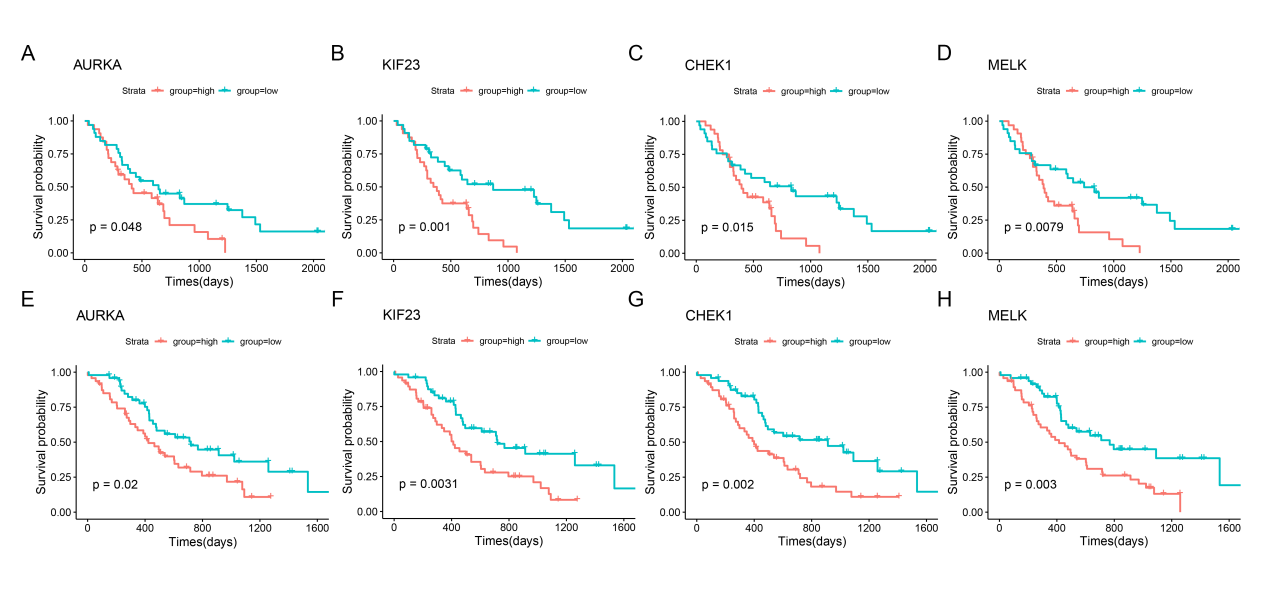


**Supplementary Figure 4 |** Survival analysis of GSE62452 and ICGC data sets

In order to verify the prognostic value of the 4 key genes in PDAC, we used GSE62452 data set with 65 cases of complete clinical information to verify our results. The results show that (**A**) AURKA; (**B**) KIF23; (**C**) CHEK1; (**D**) MELK are significantly related to OS, and the high expression group is often accompanied by poor OS. At the same time, the same results are obtained in ICGC database for these 4 genes (**E**) AURKA; (**F**) KIF23; (**G**) CHEK; (**H**) MELK. P-value in the plots is obtained by log-rank test.


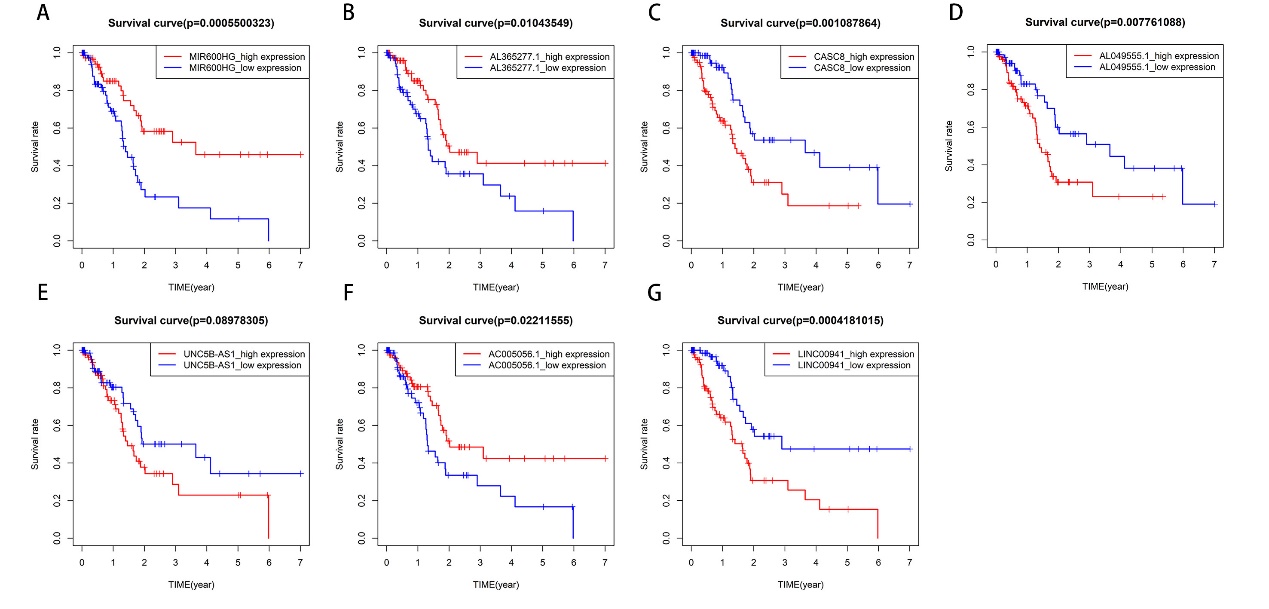


**Supplementary Figure 5 |** Description of the K-M survival curves of 7 lncRNAs

The K-M curves of 7-lncRNA signature, according to the median of gene expression, TCGA data set was divided into high expression group (red) and low expression group (blue).
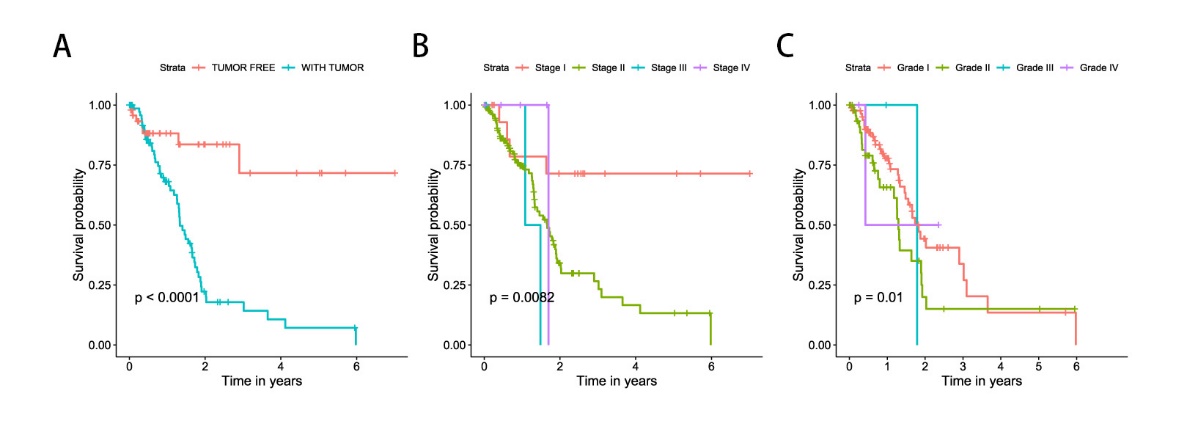


**Supplementary Figure 6 |** Description of the survival analysis results using clinical information

Kaplan-Meier curve of the clinical features for OS of the patients in PDAC data set: (A) cancer status, (B) Stage and (C) Grade. The clinical information related to OS obtained by multivariate Cox analysis, grouped according to the clinical information, and the survival curve was drawn. The patients without tumor or in early stage/grade had significantly longer survival period.

## Supplementary Tables

**Supplementary Table S1 |** Expression of DEmRNAs, DEmiRNAs and DElncRNAs
